# Gentlemen seek harmony but not uniformity: The heterogeneity of entrepreneurial team and organizational resilience

**DOI:** 10.3389/fpsyg.2022.948708

**Published:** 2022-10-13

**Authors:** Tingting Shan, Xiaoya Tian

**Affiliations:** School of Management, Nanjing University of Posts and Telecommunications, Nanjing, China

**Keywords:** the heterogeneity of entrepreneurial team, cross-boundary search, organizational resilience, Confucian traditional culture, the framework of “structure-behavior-result”

## Abstract

The purpose of this research is to investigate the association of the heterogeneity of entrepreneurial team with organizational Resilience. In an uncertain environment, whether new ventures can form entrepreneurial resilience at the organizational level in adverse events becomes the key to sustainable development. Based on the theory of heterogeneous advantage and identity characteristics, this manuscript constructed a research framework of “Structure-Behavior-Result” and described the mechanism and boundary conditions of the heterogeneity of entrepreneurial team affecting organizational resilience in detail. The role of Confucian traditional culture as a moderator has also been analyzed. Data has been obtained from 390 entrepreneurs in China. All hypotheses were tested using moderated mediation model. It has been found that the heterogeneity of entrepreneurial team has positive effect on organizational resilience. It has also been discovered that cross-boundary search behavior acted as a partial mediator between the heterogeneity of entrepreneurial team and organizational resilience. The Confucian traditional culture strengthens the relationship between them. The results are helpful in understanding the internal mechanism of the heterogeneity of entrepreneurial team affecting organizational resilience. Theoretical and practical implications have been highlighted and future research suggestions have been provided.

## Introduction

The sudden outbreak of COVID-19 has brought huge volatility to the global economic and political landscape (Hadjielias et al., 2022; Hajishirzi et al., 2022; Heinze, 2022; Lapoutte and Alakpa, 2022). The volatility of the external environment is the result of all kinds of long-term and short-term risks (Grassi and Nicole-Berva, 2022). In this environment, the development path and business model of enterprises are strongly impacted (Xie et al., 2022). Innovation is of great significance to national economic development (Do et al., 2022), and entrepreneurship is the concentrated embodiment of innovation (Yuan et al., 2022). New ventures have obvious disadvantages of resource deficiency and lack of legitimacy (Sheikhrabori et al., 2022). As a result, they face more challenges and risks and a higher rate of entrepreneurial failure (Khanzad and Gooyabadi, 2022). Nowadays, the external environment is characterized by variability, uncertainty, complexity, and ambiguity (Lawson et al., 2022). Under such normal circumstances, whether new ventures can form entrepreneurial resilience at the organizational level in the impact of adverse events becomes the key to sustainable development (Jang et al., 2022). The concept of resilience originated from physics and was later introduced into ecology, engineering, psychology, and management (Kunz and Sonnenholzner, 2022). Resilience means “rebound,” “bounce back,” “inverse resistance,” and “resilience” (Raver Luning and Ledford, 2022). Organizational resilience is the ability of teams to function effectively in the face of entrepreneurial adversity, stress, and uncertainty (Ewertowski and Butlewski, 2022). It is the ability and process of effectively coping with and actively adjusting to changes, adversities, and disturbances caused by entrepreneurship (Al-Matari et al., 2022). It is the key element of entrepreneurial success and has great significance and value for entrepreneurial theory research and mass entrepreneurship practice (Deason et al., 2022). Organizational resilience is a structural and procedural power that enables an organization to eliminate pressure (Akpan et al., 2022), maintain cohesion (Li and Gelfand, 2022), recover from setbacks (Yang, 2022), and effectively cope with crisis management (Alves et al., 2022). Kim and Okazaki (2022) regarded high stability as a core feature of organizational resilience. Organizational resilience is not static. Change, adversity, or interference is the premise of the existence of team organizational resilience (Chai et al., 2022; Yang et al., 2022). Organizational resilience is impossible without change, adversity, or interference (Cz et al., 2022). Adapting and growing is an important part of the result of resilience (Wagner et al., 2022). Considering that the formation of organizational resilience is a long-term process and subject to the scarcity of research context (Beylier and Fortuné, 2022), most of the current research on organizational resilience focus on mature enterprises (Tedeschi et al., 2022). Most studies on the growth of new ventures focus on entrepreneurial resilience at the individual level of entrepreneurs (such as optimism, tenacity, and self-improvement) (Cezar et al., 2020; Nishioka, 2022). At the same time, less attention is paid to entrepreneurial resilience at the organizational level of new ventures that are weak in risk resistance (Choi, 2020). In the context of crisis, we need to reflect on the cultivation and improvement of entrepreneurial resilience at the organizational level of new ventures (Chen et al., 2020; Guo, 2020). This is also an important issue that academic and practical circles need to pay attention to. Research has shown that, amongst developing economies, China has a larger segment of entrepreneurs who have contributed to significant economic progress (Annarelli and Nonino, 2016). Further, expansion of the entrepreneurship sector in China’s economy is an important driver of sustainable economic growth (Barasa et al., 2018). These facts make China the right context for fruitful entrepreneurship research.

The research on the heterogeneity of entrepreneurial team mainly refers to the existing research framework of TMT heterogeneity (Meyer et al., 2020; Simone and Thiele, 2020; Rumyeni et al., 2021; Suwanprasert, 2021). Based on higher-order theory, many scholars recognize that founding team heterogeneity plays an important role in organizational growth (Gerlitz et al., 2020; Liang, 2020). Localization is an important direction for the expansion of the heterogeneity of entrepreneurial team theory (Paquay et al., 2021) and organizational resilience theory (Liu et al., 2020). The selection of mediating variables and moderating variables in existing studies is relatively homogeneous with western theories (Xu, 2021). This selection of mediating variables and moderating variables is not conducive to the guidance of theory to practice (Jing et al., 2020). This choice also led to a divergence in the findings of the study (Clement et al., 2021). Therefore, to uncover the “black box” formed by the organizational resilience of new ventures in the context of Localization in China, it is necessary to consider the characteristics of local culture (Arikan et al., 2020) and interpret the mediating and moderating effects from a new perspective (Muguto and Muzindutsi, 2022). Considering the long-standing problems of culture and economy (Niedermeyer et al., 2020), Weber’s debates on culture and economic ethics, new institutional economics (Carnevali et al., 2021), and informal institution theory (Fricke et al., 2022) have made important contributions to studying the relationship between culture and economy (Clark and Sourdin, 2020). With enterprises as the main body of economic development, how culture affects the growth of enterprises has attracted more and more scholars’ attention (Abudureheman and Nilupaer, 2021). Under the current background of global cultural convergence (Lutkova, 2020), does Traditional Chinese Confucian culture still influence national economic development and enterprise growth (Yin et al., 2020)? Generally speaking, enterprises have the characteristics of local embedment (Gao et al., 2021), so they are easily influenced by local social culture (Ion, 2015). Local cultural capital is the unique endowment of production factors of enterprises. As the basic unit of industry, the growth of enterprises is closely related to local culture (Sebenius, 2002). In traditional Chinese culture, Confucian culture is the main body and essence (Foy et al., 1997). Given the important research value of Confucian culture, many scholars explore the economic consequences of Confucian culture from different perspectives (Vanaelst et al., 2010; Zheng et al., 2015; Wang et al., 2019). At the macro-level, Confucian culture plays a positive role in promoting economic growth (Xie et al., 2011), modernization process (Chen and Hao, 2008), and legal construction (Xie and Gang, 2009). At the micro level, some studies have pointed out that Confucian culture plays an important role in corporate behavior decisions such as enterprise innovation (Vogel et al., 2014), employee employment security (Schjoedt et al., 2013), internal control (Khan et al., 2014), corporate risk-taking (Bjrnli and Aspelund, 2012), and corporate charitable donation (Zhou et al., 2015). Existing literature has found that culture has a profound impact on corporate behavior (Ganotakis and Love, 2012). Still, no literature has studied the moderating effect of Confucian culture, and there is a lack of systematic analysis of Confucian culture (Schjoedt and Kraus, 2009) and the organizational resilience of new ventures (Dufays and Huybrechts, 2015). Confucian culture is crucial to the growth and organizational resilience of Chinese companies (Jin et al., 2016). As an informal system, Confucian culture can make up for the limitations of the current Chinese formal system (Leary and Devaughn, 2013). The effect mechanism of Confucian culture on organizational resilience of ventures is still unclear and needs to be further studied (Chen, 2008). Under the current situation that the formal system has not been perfected (Li and Li, 2009), how to alleviate the inherent weakness of ventures with the help of the informal system of Confucian culture (Wu et al., 2010), improve organizational resilience (Yu and Man, 2009), and promote the growth of enterprises (Marylyn et al., 2013) has become an urgent issue to be solved. In this field, there is a lack of understanding of whether the associations hold in developing economies (Evran et al., 2014), so more research is required. More research is also needed about the impact of the heterogeneity of entrepreneurial team on organizational resilience (Anuar and Juri, 2013). This is why there are calls for studies assessing how the heterogeneity of entrepreneurial team impact organizational resilience (Carden et al., 2018; Duchek, 2020).

Because of this, based on the theory of heterogeneous advantage (Groh and von Liechtenstein, 2010), this study attempts to construct a “structure-behavior-result” research framework. This manuscript intends to answer the following questions. How does the heterogeneity of entrepreneurial team affect organizational resilience? By what mediating mechanism is the effect transmitted? Is there a boundary condition for the mediating agent? Considering that organizational behavior of enterprises is affected by cultural context (Studdard, 2004), this study introduces the degree of influence of Traditional Confucian culture on new enterprises, a special group. And taking the influence degree of Confucian traditional culture as a moderating variable. Therefore, this paper can describe the mediating effect of cross-border search behavior on the relationship between the heterogeneity of entrepreneurial team and organizational resilience. And explore the boundary conditions of this mediation. Therefore, it provides practical enlightenment for new enterprises to cultivate and improve organizational resilience.

The marginal contribution of this paper includes the following aspects. Firstly, this manuscript is helpful in expanding the research on traditional Confucian culture and organizational development in China (Park and Rho, 2016). It is of great significance to carry forward Confucian culture and advocate Confucianism to govern enterprises. The government has repeatedly supported promoting traditional Chinese culture and enhanced cultural awareness and confidence. The research conclusions of this paper provide empirical evidence for the Confucian culture in optimizing ventures corporate governance and improving organizational resilience. China is a relevant context here because it is one of the world’s largest developing economies (Eskreis-Winkler et al., 2014). It had a deep-rooted denunciation of private enterprise, which started changing in the 1990s and now the country has a strong private sector that provides multiple opportunities for entrepreneurship (Eskreis-Winkler et al., 2014). However, the country still has a low level of entrepreneurship so research is needed in this area (Fisher et al., 2019). The key research objectives of this study are to explore influence of the heterogeneity of entrepreneurial team on organizational resilience. Moreover, this article also helps in reducing people’s resistance and prejudice against traditional Confucian culture. More importantly, this paper provides practical enlightenment on how to use Confucian traditional culture to cultivate and enhance organizational resilience of new ventures in the context of frequent adverse events in China (Dheer and Salamzadeh, 2022). Secondly, refine the boundary of the impact of the heterogeneity of entrepreneurial team on organizational resilience. This refinement not only reconciles the differences of previous studies, but also to deepen people’s understanding of how the heterogeneity of entrepreneurial team affects organizational resilience.

## Theoretical basis and hypothesis

The formation process of organizational resilience is “ironing out” the risks and uncertainties brought by internal and external shocks (Ge et al., 2021; Gu and Liu, 2021; Wu et al., 2021). The focus of organizational resilience research has been shifting with the change in crises (Sappor, 2021). Flexibility emphasizes “rapid adaptation to environmental changes” (Pham and Pham, 2021). Agility focuses on “quickly identifying opportunities (Percy and Dow, 2021), changing direction (Kim et al., 2021), and avoiding conflict” (Han, 2022). Robustness is more concerned with maintaining function in the face of interference. In contrast, the organizational resilience of new ventures emphasizes the ability of the organization to recover and rebound under the impact of adverse events and grow against the trend in the process of reflection and improvement (Gölgeci and Kuivalainen, 2020). Although flexibility, agility, and robustness are structurally similar to resilience, the former is necessary to deal with everyday problems and changes. In contrast, resilience is seen as an essential success factor in coping with unexpected threats and responding to crisis changes (Koh and Ong, 2021). Organizational resilience is an extension of the concept of resilience in ecology and physics (Zhang, 2021), used initially to measure a system’s ability to recover after a disaster (Roker, 2021). The multi-dimensional and multi-level characteristics of organizational resilience make scholars’ interpretation of its concept gradually enter the “jungle state.” Representative literature, such as Huang (2007), pointed out from the perspective of ecology that organizational resilience is to re-establish a balanced adaptive behavior between the organization and the existing environment. Khodakovsky et al. (2020) constructed the conceptual framework of organizational resilience from the perspective of systems science and believed that organizational resilience results from the combined effects of the three elements of corporate position perception, situational integration, strategy formulation, and execution. From the perspective of crisis management, The Domestic scholar Kristinsson et al. (2016) defined organizational resilience as the ability of an organization to reconstruct organizational resources, processes, and relationships in a situation, recover quickly from the crisis and realize countertrend growth by using the crisis. It is not hard to find that despite the diverse perspectives of domestic and foreign scholars, the view that organizational resilience is essentially a “capability” has been widely accepted. In this study, organizational resilience is considered a kind of implicit “stock” + “incremental” capability. From the perspective of capacity, organizational resilience is divided into organizational resilience and organizational integration and optimization capability. Organizational resilience refers to the stock capacity of organizational adaptability that grows and develops over time. Organizational integration and optimization capability refer to the incremental ability of an organization to predict the next crisis and adjust its strategy rapidly.

The relationship between the heterogeneity of entrepreneurial team and team performance is the focus of research on the heterogeneity of entrepreneurial team (Tevera, 2020). According to most current research results, excessive team heterogeneity hurts organizational decision-making efficiency, organizational innovation climate, and organizational performance or presents an inverted “U” shaped relationship. In general, the management field has conducted extensive empirical studies on the direct effect of the heterogeneity of entrepreneurial teams on team performance and the impact of different types of fracture zones on team performance through various mediating variables and moderating variables from the perspectives of general and contingency. Early studies focused on the direct effect of the heterogeneity of entrepreneurial team on team performance from a broad perspective. For example, Orth and Schuldis (2021) and Rucai (2021) believed that the heterogeneity of entrepreneurial team would lead to team conflict, intensify distrust among members and reduce group satisfaction. Voghera and Giudice (2019) and Thanh (2012) investigated the impact of the heterogeneity of entrepreneurial team of TMT on team performance by taking the characteristics and attributes of TMT members as a whole. They concluded that they presented an inverted “U” shaped relationship. The effect mechanism of the TMT the heterogeneity of entrepreneurial team on team performance is highly complex. It is difficult to draw a definitive conclusion only considering the correlation between TMT the heterogeneity of entrepreneurial team and team performance by ignoring the heterogeneity of the research object and the specific research context. The neglect of this relationship is the root cause of the divergence in academic circles on the relationship between TMT the heterogeneity of entrepreneurial team and team performance for a long time. Therefore, the general perspective of the utility study of the heterogeneity of entrepreneurial team has gradually changed to the contingency perspective. Entered the stage of contingency perspective, academic circles based on the heterogeneity of entrepreneurial team with the team task-related degree, the team fault classification for gender, age, race is characterized by the demographic attributes, such as the social attribute of the heterogeneity of entrepreneurial team. It is characterized by education background, work experience of information related to the heterogeneity of entrepreneurial team, or according to physiological characteristics of the heterogeneity of entrepreneurial team and task-oriented the heterogeneity of entrepreneurial team. Combined with the mediation and regulation model, this paper discusses. Such studies refine the types of independent variables in the heterogeneity of entrepreneurial team of the senior management team, laying a foundation for exploring the complex and comprehensive mechanism. Even from the perspective of contingency, there are still differences in the action direction of the heterogeneity of entrepreneurial team in the academic circle.

At present, there are mainly the following viewpoints: (1) Resource view. Scholars believe that the high-strength fracture zone of the team brings heterogeneous resources to the organization, produces resource redundancy, and improves the strategic and financial flexibility of the enterprise, which is beneficial to the team performance (Cheng, 2021). However, they ignore the development stage of the enterprise. The life cycle of an enterprise includes venture period, development period, maturity period and decline period. The intensity of the heterogeneity of entrepreneurial team in different time periods may bring different utility. (2) Risk view. Scholars who hold such ideas believe that the high-strength fracture zone will cause low decision-making efficiency and a lack of organizational identity within the organization, which will make enterprises struggle in the fiercely competitive market environment (Carleo, 2021). However, the internal action path has not been deeply described. (3) Neutral view. Scholars with a neutral view realize that the relationship between the heterogeneity of entrepreneurial team and team performance needs further consideration of firm characteristics, mediating mechanism and boundary conditions (Carleo, 2021).

Sociologists divide the connections between network nodes into affective and task-oriented links (Karlsson et al., 2017; Vihervuori, 2017). Homogeneity leads to more robust (Amaya and Liao, 2017) and higher performance links based on emotional orientation. Homogenous combinations can strengthen links, which is called robustness (Perera, 2018). In task-oriented linkage, heterogeneity is more significant (Aga et al., 2016), conducive to the organization’s access to a broader range of opportunities and resources (Omri and Boujelbene, 2021) and the completion of more challenging tasks (Ensley, 1997). Heterogeneous cooperation to meet challenges is called progress (Ensley et al., 1998). According to the theory of heterogeneous advantage (Lechler, 2001), within an organization, senior management teams with different backgrounds can view crisis events from different perspectives (Yee et al., 2021), resulting in higher decision-making efficiency and stronger problem-solving ability (Kelly et al., 2021). This multi-angle approach brings higher decision-making efficiency and stronger problem-solving ability to the organization (Haryanto et al., 2021). This effect also exists between organizations (Yee et al., 2021). Two organizations with diverse technical and industry backgrounds will achieve better innovation output when they jointly develop projects, confirming the positive effect of cross-border search (Payne et al., 2021). Enterprise resilience is essentially the richness of the network established between cognitive nodes (Le et al., 2021). It is recognized by the academic community that node connection starts from the asymmetry and exchange of information and resources (Liu J. H., 2021). This feature of node connection makes the information overlap degree of the interconnection of homogeneous nodes high (Lee, 2021). Heterogeneous node interconnection spans other information sources (Bennett, 2021) and transfers information resources that do not belong to the organization where the node is located to different nodes, forming an “information bridge” (Jiang and Wei, 2021). The information niche of the enterprise organization is promoted and occupies more and more important and diversified “structural holes” (Liu X., 2021). Entrepreneurial team members with the heterogeneity of entrepreneurial teams are initially heterogeneous individuals which are matched to form complex heterogeneous interconnection under the framework of the same task orientation (Eggert and Roetz, 2022) and become an integrated complex network system to counter the uncertain market environment and technological environment (Vo and Nguyen, 2021). Entrepreneurial team fault lines drive connections between unique cognitive nodes, resulting in more creative and high-performance organizational structures (Zhao, 2021).

### The heterogeneity of entrepreneurial team and organizational resilience

Entrepreneurial Team heterogeneity refers to the differences in demographic characteristics such as age, sex, educational background, functional background, and vocational skills such as entrepreneurial experience, industry experience, some scholars also bring the difference of social capital into the connotation of entrepreneurial team Heterogeneity, such as the difference of cognition and their own relationship network (Fan, 2021). To some extent, these differences reflect the difference of entrepreneurial team members in perception level, behavior choice, value judgment, and so on, which directly affects the entrepreneurial team’s behavior, and then affects the organizational resilience of the enterprise. At present, scholars worldwide have different views on the relationships of the team heterogeneity and organizational resilience. Some scholars start from the theory of information decision-making, hold that team members with high heterogeneity are conducive to the sharing of knowledge and information within the team and organizational decision-making (Karlsson, 2021; Roker, 2021). According to the theory of social cognition and team cooperation, other scholars believe that the higher the degree of Heterogeneity of team members, the greater the negative effects of cognitive divergence and goal conflict between teams, thus affects the team member overall cooperation level and the efficiency (Bika and Kalantaridis, 2019). This study argues that these differences are due to the fact that scholars do not consider the life cycle of a business, ignoring whether the business is a venture or a mature enterprise (Wang and Lin, 2019; Lee, 2021). Because there are many differences between ventures and mature enterprises. This study believe that entrepreneurial team heterogeneity has a positive impact on organizational resilience in venture enterprises, mainly through the following three mechanisms: ➀ Highly Heterogeneous Ventures teams get information more quickly. Teams with high heterogeneity usually have multiple information channels, and multiple and rich information can enable new enterprises to be exposed to the changes of the external environment in the shortest time, promote the occurrence of cross-border search behavior, and adjust the action strategy in time. ➁ The entrepreneurial team with high heterogeneity has strong information processing ability, and the members with high heterogeneity have different market experience and perspective of view, it determines that the whole entrepreneurial team has a multi-dimensional perspective, which is helpful to the screening and screening of decision-making information. ➂ The entrepreneurial team with high heterogeneity has high decision-making efficiency, and the team members with high heterogeneity have diverse life experience and employment experience, such teams tend to have a high level of creativity and a high degree of complexity in developing strategies for action. Compared with homogeneous teams, diversified teams are more likely to launch a large number of large-scale, multi-type actions, which will help to improve the organizational resilience. In the highly uncertain external environment, the members of the entrepreneurial team are the main decision-makers and the core of allocating the strategic resources of the organization, its heterogeneous resources of human capital and social capital not only open up a shortcut for the rapid production recovery, but also provide the possibility for the sustainable growth of the organization.

Therefore, this manuscript proposes the following hypotheses:

H1: The heterogeneity of entrepreneurial team has a positive impact on organizational resilience.

H1a: The heterogeneity of entrepreneurial team has a positive impact on the adaptive recovery ability.

H1b: The heterogeneity of entrepreneurial team has a positive impact on the integrated optimization ability of the organization.

### The mediating role of cross-border search

Resource-based theory explains that the Intrinsic Motivation for an organization to acquire unique competitive advantage resources lies in the complementary flow of diversified knowledge within and outside the organization, which fully reflects the importance of cross-border search to organizational resilience. Cross-border search will make enterprise decision-making more comprehensive and rational, enhance the cognitive dimensions of entrepreneurial team members, multi-dimensional cognitive technology environment, market environment. In adverse situations, the members of the ventures team will consider the long-term development of the enterprise and make a more comprehensive and reasonable emergency plan to turn the crisis into an opportunity. Cross-border search can increase the possibility of obtaining diversified information, thus improving the crisis bearing capacity of an organization.

The occurrence of any behavior is inseparable from motive, premise, and foundation (Liu J. H., 2021). Cross-border search behavior is a kind of search activity under dynamic environment (Lin, 2021). In cross-border search behavior, organizations cross existing institutional boundaries and knowledge boundaries in order to obtain heterogeneous resources (Zhou, 2022). The heterogeneity of entrepreneurial team activates unique cognition (motivation), generates new needs (premise), and forms new connections (foundation) for the organization, which promotes the occurrence of cross-border search. Firstly, the entrepreneurial group has a high fracture zone strength, which means that the resources within the team are heterogeneous, and the cognition is diversified. The effect of “1 + 1 > 2” can be formed when different cognition is superimposed (Niedenführ, 2021). This effect activates new knowledge that would otherwise go unrecognized or ignored in the group. The new cognition’s demand for more diverse and heterogeneous resources is stimulated, which forms a strong motivation and an essential prerequisite for cross-boundary search from the subjective aspect of the team. The theory of information decision making holds that decision making is a process of informed generation, integration, and regeneration, and information is transmitted between information source and decision-maker (Shi, 2021), thus affecting decision-making behavior. Boundary-crossing behavior is a kind of non-procedural decision, which depends on the accumulation of career experience of entrepreneurial team members (Lu, 2022). The cognition of entrepreneurial team members is based on the limited rationality of people, and the cognitive traits of the team have a crucial influence on the decision-making of the entrepreneurial team, which is the key to the sustainable growth of the entrepreneurial team. Secondly, the heterogeneity of entrepreneurial teams in the early stage of entrepreneurship can prevent the group from falling into the “team myth” and reduce the risk of collective decision-making. Members of a multi-background entrepreneurial team can also bring recognition from investment institutions with different backgrounds to the enterprise (Mokline and Abdallah, 2022). Thirdly, the occurrence of cross-border search depends on whether the entrepreneurial team can subjectively break down various organizational barriers. It also depends on whether there are objective conditions to ensure the integration of resources, knowledge, and capital advantages. According to the social capital theory, the diversity of entrepreneurial teams (the higher the fault line, the greater the diversity) can provide rich, comprehensive, high-quality information, and broader social connections for the transaction integration and other related decisions in cross-border behaviors (Geschwind et al., 2022). Entrepreneurial teams with high the heterogeneity of entrepreneurial teams or high heterogeneous backgrounds have advantages in social capital accumulation and expansion (Bajgori et al., 2022).

This advantage helps in providing diversified perspectives, and resources for the sustainable development of entrepreneurial teams (Percy and Dow, 2021). More importantly, a new connection with greater breadth, depth and centrality is formed externally, which undoubtedly provides an objective basis and guarantee for cross-border search behaviors and is conducive to entrepreneurial teams’ exploration of organizational boundaries and cross-border behaviors. Finally, and more importantly, the heterogeneity of entrepreneurial team can help avoid the “dominance trap.” Organization management theory emphasizes the play their advantages. If an organization’s most significant advantage is “hammer,” then look at all the questions are regarded as “nails,” but in the digital age, many problems do not have a “nail” properties, these questions have no ready-made solutions “hammer,” if the organization takes advantage of thinking at this moment, is easy to fall into “advantage trap.” The existence of the heterogeneity of entrepreneurial team enables the organization to “see the screw, put down the hammer and find the screwdriver.” This process not only helps organizations treat new problems with caution, but also helps organizations make objective judgments and expand their toolbox. Therefore, it has a positive effect on enterprises’ cross-border search behavior.

Cross-border search behavior on organizational resilience is mainly reflected in three aspects. Firstly, cross-border search is an essential corporate learning mechanism, providing heterogeneous resources (Villa, 2021). The resource-based theory believes that unique, scarce, and difficult to imitate resources are the source of an entrepreneurial team’s competitive advantage and can create continuous value for the entrepreneurial team. New venture teams have problems such as new entry defects and too small scale and are severely constrained in obtaining resource information from outside (Wang and Wang, 2021). Organizational learning theory holds that learning communication is a crucial way to drive resource acquisition, absorption, and application (Su et al., 2021), the learning is also an essential mechanism of resource accumulation in entrepreneurial teams of new ventures (Salamzadeh and Dana, 2022). Cross-border search enables entrepreneurs to absorb relevant experience and knowledge. At the same time, entrepreneurs with cross-border solid searches tend to be open-minded and thirsty for skills, which helps attract professional talents to join in. Communication and learning between organizations can improve their financial management level and the profitability of entrepreneurial teams, which is conducive to gaining the trust and support of financial institutions and venture capital institutions. Entrepreneurial teams with intense cross-border search ability can more accurately grasp the national policy orientation, to obtain more preferential policies. Secondly, the cross-border search behavior improves the opportunity capacity of the organization. There is a constant exchange of resources between the ventures team and the outside world (Ge et al., 2021). This exposes entrepreneurs to a wealth of new ideas and knowledge. The stronger the opportunity detection ability of the organization, the easier it is to identify and obtain the required information, technology, knowledge, and legitimacy resources. Learning new marketing models and other successful business experiences from network members can also help the ventures team to obtain more marketing resources. The stronger the corporate opportunity coordination ability is, the more tacit knowledge entrepreneurial teams acquire through the relationship network, the higher the efficiency of resource accumulation, and the higher the organizational resilience. Finally, cross-border search breaks organizational path dependence. Entrepreneurial teams will form capability rigidity and path dependence due to the reuse of inherent knowledge (Zani, 2022). Boundary-crossing behavior can break down this capacity rigidity and path dependence, and it can effectively push innovation boundaries. But it also brings new challenges to the value judgment and resource integration decision of the entrepreneurial team (Zhang and Chan, 2021). Therefore, this paper proposes the following hypotheses:

H2: Cross-border search plays a mediating role between the heterogeneity of entrepreneurial team and organizational resilience.

H2a: Cross-border search plays a mediating role between the heterogeneity of entrepreneurial team and adaptive recovery ability of the organization.

H2b: Cross-border search plays a mediating role between the heterogeneity of entrepreneurial team and integrated optimization ability of the organization.

### The moderating role of Confucian traditional culture

Although the heterogeneity of entrepreneurial teams can provide the cognition of diversified perspectives for organizations, the diversified perspectives also need to be stimulated and discussed based on the principle of integration (Newton-Levinson et al., 2022). Discussing the same principle from different perspectives is the “gentleman of harmony without uniformity” advocated in the traditional Confucian culture (Henderson et al., 2022). The Traditional Confucian culture delimits a vital boundary between the heterogeneity of entrepreneurial team and enterprise growth (Ewertowski, 2022). That is, only under the cognitive framework of integration the heterogeneity of entrepreneurial team will play a positive effect otherwise it will inevitably lead to the disintegration of the organization, not to mention the sustainable development of the organization. Based on this, it is concluded that Confucian traditional culture plays a vital role in regulating the relationship between the heterogeneity of entrepreneurial team and organizational resilience.

The promotion effect of the heterogeneity of entrepreneurial team on cross-border search behavior is mainly realized by stimulating new cognition (Navaei et al., 2022), generating unique needs (Lyng et al., 2022), and forming new connections (Jo and Lee, 2022). In this process, the effectiveness of the heterogeneity of entrepreneurial team is highly dependent on the cultural environment (Lehner et al., 2022). First of all, the traditional Confucian culture of “respecting teachers and valuing education and talent orientation” has strengthened the team’s new demand for talents and talents inspired by the heterogeneity of entrepreneurial team, making the organizational subjective will of cross-border search stronger. The Confucian cultural thoughts of “getting knowledge from things” and “honesty and sincerity” reflect the cultural atmosphere of thirst for knowledge in ventures. Ventures with a high degree of Confucian traditional culture attach more importance to the pursuit of human capital and wisdom. “Wisdom” is one of the five pillars of Confucian culture (Alibašić, 2022). The influence of Confucian culture means that entrepreneurial team members attach importance to their own and employees’ talent and wisdom, which can effectively stimulate the cross-border search behavior of “all rivers are boundless” at the organizational level. Secondly, Confucian culture advocates “moderation.” Moderation means impartiality, the acceptance, and integration of different cultures (Helmold et al., 2022). This “inclusive culture” improves the team’s new awareness of resources, opportunities and strategies inspired by the heterogeneity of entrepreneurial team, making cross-border search behaviors more smoothly. Finally, Confucianism believes that “harmony is the best way to achieve the world,” righteousness before benefit is glorious, benefit before justice is a disgrace, emphasizes philanthropic motivation, and believes that “harmony without uniformity” is conducive to the realization of win-win. The generous culture in the Confucian traditional culture expands the social capital connection needed in cross-border search inspired by the heterogeneity of entrepreneurial team and provides a more solid external guarantee for the cross-border search behavior.

Further analysis, Confucian culture holds that “all the streets are full of saints” and “everyone can learn from Yao and Shun.” Influenced by such a “harmonious spirit,” ventures tend to be more inclined to “harmony without uniformity” when making business decisions and treat the cognition of diversified entrepreneurial teams with a more tolerant attitude. As an informal system, Confucian culture has alternative governance values to the formal system. Compared with the rules of organizational governance, the idea of “three people in a company must have my teacher” is more conducive to promoting the team to actively absorb and internalize heterogeneous resources of different members and form internal resource redundancy. The cognitive brand solidified by the Confucian culture of “anticipating danger in times of peace” will also strengthen the motivation of organizations. The concept of “going with The Times” endows the entrepreneurial team with the idea of contingency, sizing up the situation, constantly crossing boundaries, and actively adjusting the pace of innovation and entrepreneurship. All these are more conducive to the formation and improvement of organizational resilience (Zhai, 2018).

In a word, the existence of the heterogeneity of entrepreneurial team will make the connections between entrepreneurial team members extremely fragile in ventures with weak Confucian traditional culture influence (Ribeiro et al., 2022). Even though the heterogeneity of entrepreneurial team positively affects cross-border search behavior and firm resilience growth, the persistence of such positive effects is low (Hussain and Papastathopoulos, 2022). On the contrary, in the new ventures influenced by Confucian traditional culture or deeply infiltrated, the entrepreneurial team members uphold the concept of “harmony without uniformity” (Erwin et al., 2022). Therefore, the association between members is stronger and more durable, and the heterogeneity of entrepreneurial team has a stronger and more significant effect on cross-border search behavior and organizational resilience.

Therefore, this manuscript proposes the following hypotheses:

H3: Confucian traditional culture has a positive moderating relationship between the heterogeneity of entrepreneurial team and cross-border search.

H4: Confucian traditional culture has a positive moderating relationship between the heterogeneity of entrepreneurial team and organizational resilience.

H4a: Confucian traditional culture has a positive moderating relationship between the heterogeneity of entrepreneurial team and adaptive recovery ability of organization.

H4b: Confucian traditional culture has a positive moderating relationship between the heterogeneity of entrepreneurial team and integrated optimization ability of the organization.

**FIGURE 1:**
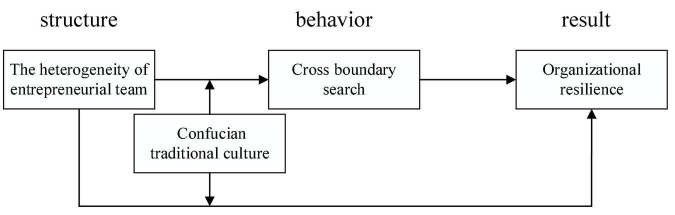
“Structure-behavior-result” theoretical model.

Based on all of the above proposed hypothesis and the theoretical foundation the conceptual association among variables is presented below in Figure 1.

## Materials and methods

### Sample selection and data collection

**FIGURE 2:**
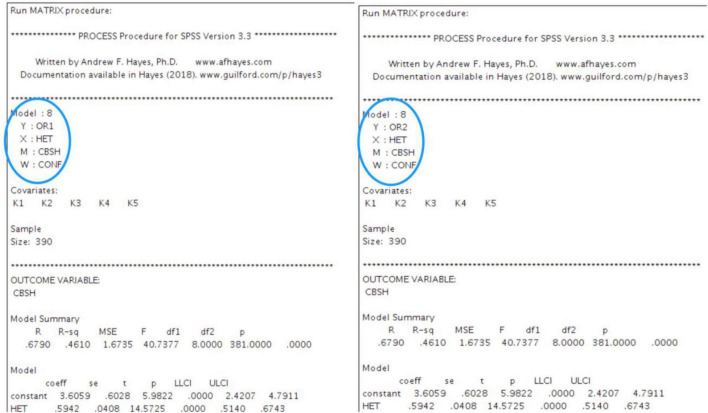
Screenshot of the running interface of the software used in this study.

Data were collected from the owners of entrepreneurial ventures located in China. The present study was directed using a moderated mediation model (Vogel et al., 2014). The primary reason of choosing the members of entrepreneurial team from entrepreneurial ventures for data collection was to deeper explore the influence of the heterogeneity of entrepreneurial team on organizational resilience (Carleo, 2021). The core variables in this study are all at the organizational level, and two conditions are strictly controlled when sample enterprises are selected: The enterprise has been established within 5 years and is in good operating condition (Su et al., 2021); The team should have at least three members (Do et al., 2022). Considering the data availability, this study selected 510 ventures nationwide in China, which are distributed in apparel production, agricultural breeding, intelligent manufacturing, new materials, service consulting, biomedicine, and other fields. Based on validated scales, we built a preliminary questionnaire. We altered and merged these measures before conducting the final survey. Thirty-two randomly selected members of entrepreneurial team in China, completed the pilot questionnaire and checked its layout as well as content validity. We constructed the final questionnaire to improve the instrument’s relevance and readability. The cross-sectional questionnaire included 23 items, based on the pilot survey and observations of Chinese language expression patterns. The software used in this study is PROCESS procedure for SPSS3.3, and the running interface is shown in Figure 2.

To ensure the quality of the research results and smooth process, the research group communicated with each other over the phone to indicate the intention of the research. We contacted the entrepreneurs and presented our research purpose for obtaining permission to conduct the study. Then make an appointment with the members of entrepreneurial team to fill in the questionnaire. Due to the epidemic, telephone calls, Tencent meetings, and other methods were selected for full communication, so that respondents can fill in the questionnaire more conveniently. This study used two ways to reduce the common method bias. ➀ During transmission, the subjects were told that the research data were only for academic research, and there was no difference between right and wrong in all answers. They also promised to keep the obtained data strictly confidential, dispel the psychological concerns of the subjects, and do an excellent job of psychological isolation, so that the issues could answer the questions more accurately. ➁ Collect data in two-time points (3–4 weeks). Each questionnaire is divided into parts A and B. Three founders are randomly selected from each team to fill in section A. After an interval of 3–4 weeks, the other founders (the rest members of entrepreneurial team) were asked to fill in part B of the questionnaire. If there are more than 3 founders in the team, 2 founders are randomly selected to fill in questionnaire A and the rest members fill in questionnaire B. Each venture was numbered, and the questionnaires collected by the same enterprise were matched and merged.

The survey was conducted from September 2021 to February 2022. This study gave out 500 questionnaires, recalled 449 questionnaires, and effectively matched 436 questionnaires. After removing invalid data or answering questionnaires with significant logical errors, 390 valid questionnaires were retained and merged.

We calculated the following statistics based on demographic data. The results of descriptive statistical analysis of sample enterprises are shown in Table 1. As shown in Table 1, 50.26% of the sample enterprises are small and medium-sized enterprises. 49.74% are large-scale. This shows that the size of enterprises is evenly distributed. The proportion of enterprises with established years ranging from 2 to 4 years is the largest. 85.38% of new ventures are in the industry of machinery, electrical, electronics, communications, precision equipment (high-tech industry), accounting for the largest proportion. There were 118 ventures with annual turnover of 500,000–1 million, accounting for 30.26% of the total.

**TABLE 1:** Demographics of survey respondents.

Variable	Category	Frequency	Percentage (%)	Cumulative percentage (%)
Enterprise scale	Small and medium-sized	196	50.26	50.26
	Large-scale	194	49.74	100.00
Years of establishment	0–1	39	10.00	10.00
	1–2	70	17.95	27.95
	2–3	79	20.26	48.21
	3–4	132	33.85	82.05
	4–5	70	17.95	100.00
Industry	Consulting and Services	4	1.03	1.03
	Daily product manufacturing	38	9.74	10.77
	Machinery, electrical, electronics, communications, Precision equipment (high-tech industry)	333	85.38	96.15
	Other	15	3.85	100.00
Average annual investment in innovation	0–100,000 RMB	66	16.92	16.92
	110,000 yuan–1 million yuan	53	13.59	30.51
	1 million–3 million yuan	149	38.21	68.72
	Over 3 million yuan	122	31.28	100.00
Annual turnover	Less than 200,000 yuan	92	23.59	23.59
	200,000 yuan–500,000 yuan	65	16.67	40.26
	500,000 yuan–1 million yuan	118	30.26	70.51
	1 million–5 million yuan	72	18.46	88.97
	Over 5 million yuan	43	11.03	100.00
Total		390	100.0	100.0

### Variable measurement

Taking into consideration the focus of this study, we included only entrepreneurs in our sample. The members of entrepreneurial team who owned entrepreneurial ventures in China were selected, using a non-probability (convenience) sampling method. The sample size is 390. All items (except the number of employees) were presented on a 7-point scale, from 1 (strongly disagree) to 7 (strongly agree).

The heterogeneity of entrepreneurial team was measured using 5 items (Zhang and Zhang, 2019). Sample items were including “My team members and I have a big difference in educational background,” “My team members and I have quite different backgrounds in terms of growing up,” “There is a big difference between my team members and me in the cognition of risk and business model.” For this construct, Cronbach’s alpha was 0.986.

Cross-border search was captured using 4 items. Sample items were including “My entrepreneurial team often communicates with external institutions (scientific research institutions, peer enterprises, upstream and downstream enterprises, etc.) for information exchange,” “My ventures team often takes the initiative to build connections and participate in the external environment.” For this construct, Cronbach’s alpha was 0.978.

Organizational resilience includes two dimensions: Adaptive recovery ability and Integrated optimization ability. Among them, Adaptive recovery ability of the organization was measured through five items. Sample items were including “My team will make use of the previous accumulated experience of the enterprise to quickly respond to rapid changes in the technical environment,” “My team can quickly adapt to changes in the market environment and form coping measures.” For this construct, Cronbach’s alpha was 0.977. Integrated optimization ability was measured through 5 items. Sample items were including “My team is keen to find opportunities in adverse events and make good use of them,” “My team has been able to break out of seemingly dangerous environments and achieve unexpected new model innovations.” For this construct, Cronbach’s alpha was 0.985.

Confucian traditional culture was measured through 4 items. Sample items were including “My company is close to the center of Confucian culture in China,” “My corporate culture follows the rules of traditional Confucian culture,” “I often advocate the benefits of Confucian traditions in business.” For this construct, Cronbach’s alpha was 0.973.

#### Control variables

The control variables in this study include enterprise scale, the establishment years, industry, annual turnover, and annual innovation investment.

## Empirical analysis and results

### Reliability analysis

It is essential to check the reliability and validity of measurement tools utilized (Studdard, 2004). Reliability analysis verifies the internal consistency of the scale, that is, whether different items can measure the same content or concept independently. Cronbach’s Alpha coefficient is mainly used in this paper to investigate the internal consistency of the scale. Cronbach’s Alpha coefficient is between 0 and 1. If the α coefficient does not exceed 0.6, the internal reliability is generally considered inadequate. When the α coefficient reaches 0.7–0.8, the scale has considerable reliability. When the α coefficient reaches 0.8–0.9, the reliability of the scale is very good (Groh and von Liechtenstein, 2010). As shown in Table 2, the total reliability of this study is 0.928, greater than 0.9. Cronbach’s Alpha of all dimensions of the scale is greater than 0.9. The results show that the scale and dimensions have high reliability, good stability and consistency, and can be used for in-depth analysis.

**TABLE 2:** Confirmatory factor analysis.

Variable KMO and bartlett inspection summary					Factor loadings
	
KMO value	X^2^	Df	Sig	Cronbach’s Alpha	
0.972	15875.613	210	0.000	0.928	
**The heterogeneity of entrepreneurial team n (HET): CA = 0.986; CR = 0.930; AVE = 0.796** Source(s): Zhang and Zhang (2019)					
HET1: My team members and I have a big difference in educational background;					0.841
HET2: My team members and I have quite different backgrounds in terms of growing up;					0.844
HET3: There is a big difference between my team members and me in the cognition of risk and business model;					0.840
HET4: My team members are quite different in age;					0.840
HET5: Risk preferences for a particular strategy differed among my team members, often in conflict.					0.839
**Cross-border search behaviors (CBSH): CA = 0.978; CR = 0.901; AVE = 0.712**					
CBSH1: My entrepreneurial team often communicates with external institutions (scientific research institutions, peer enterprises, upstream, and downstream enterprises, etc.) for information exchange;					0.825
CBSH2: My entrepreneurial team often takes the initiative to build connections and participate in the external environment;					0.829
CBSH3: My entrepreneurial team has multiple channels of marketing and social knowledge to find new areas;					0.849
CBSH4: My entrepreneurial team can conduct an in-depth search and understanding of existing marketing and social services fields					0.829
**Organizational resilience (OR)**					
***Adaptive recovery ability (OR1): CA* = *0.977; CR* = *0.921; AVE* = *0.848***					
OR11: My team will make use of the previous accumulated experience of the enterprise to quickly respond to rapid changes in the technical environment;					0.758
OR12: The team can quickly adapt to changes in the market environment and form coping measures;					0.753
OR13: My team members are able to think clearly under stressful conditions, adapt to change, cope with stress, and bounce back from hardship;					0.765
OR14: My team can effectively get out of this dilemma by using existing resources and capabilities to strategically adapt to current developments;					0.780
OR15: My team has high situational awareness, reduces its vulnerability to systemic risks, and effectively absorbs, responds to, and exploits disruptive unexpected events.					0.780
***Integrated optimization ability (0R2): CA* = *0.985; CR* = *0.940; AVE* = *0.779***					
OR21: My team is keen to find opportunities in adverse events and make good use of them;					0.707
OR22: My team has been able to break out of seemingly dangerous environments and achieve unexpected new model innovations;					0.716
OR23: My team was able to take the initiative and quickly develop concrete measures to address challenges and emerge stronger from the crisis;					0.699
OR24: My team specializes in exploiting opportunities created by disruptive accidents that threaten the survival of organizations;					0.712
OR25: My team was able to leverage change effectively and even turn adverse conditions into learning and innovation opportunities to ensure continuity in the development.					0.706
**Confucian traditional culture (CONF): CA = 0.973; CR = 0.916; AVE = 0.629**					
CONF1: My team is close to the center of Confucian culture in China;					0.856
CONF2: My corporate culture follows the rules of traditional Confucian culture;					0.862
CONF3: I often advocate the benefits of Confucian traditions in business;					0.872
CONF4: My team members often learn Confucian traditional culture and are deeply influenced by it.					0.875

### Validity analysis

Validity refers to the validity of the investigation methods, means, and results (Studdard, 2004). In other words, validity refers to the accuracy of the survey method and the data obtained, that is, accuracy. The commonly used validity test methods mainly include content validity and construct validity. Among them, content validity refers to whether the design items of the questionnaire really determine the subject to be studied. Because this paper is based on the reference of existing domestic and foreign literature, there are relevant references for the grade seven scale of each research variable. After completing the questionnaire design, the author referred to the opinions of the instructor and classmates. Then the expression of the question and the structure of the questionnaire were modified to form the Firstly draft of the questionnaire. Before the formal investigation, the questionnaire was tested in a small range, and ambiguous and repetitive questions were modified and deleted. Therefore, it can be concluded that the questionnaire has good content validity. The consistency between structure and theoretical structure reflected by measurement results is structure validity. The structural validity reflects whether the experiment really measures the hypothesis theory. Exploratory factors will be used to measure the scale’s structural validity. The purpose of exploratory factor analysis is to identify several common factors that can represent the basic framework of the scale. The degree of correlation between observed variables and common factors was also reflected.

Data should pass KMO sample measure and Bartlett’s test of Sphericity before performing corresponding factor analysis (Amaya and Liao, 2017). In order to verify the partial correlation and simple correlation coefficient between variable items. When the correlation is high, the data is suitable for factor analysis. The KMO and Bartlett test results of each variable in this study are shown in Table 2.

Kiser gives common KMO metrics: KMO value above 0.9 indicates that it is very suitable for factor analysis. 0.8–0.9 is suitable 0.7–0.8 is fair; 0.6–0.7 is acceptable 0.5–0.6 indicates not suitable. Less than 0.5 is very unsuitable. As can be seen from the Table 2, KMO value is 0.972, greater than 0.9, indicating that the data is suitable for factor analysis. Bartlett’s chi-square value of sphericity test is 15875.613 (*P* < 0.01), indicating that the relationship between various items of user variables is good enough for factor analysis.

Interpret the eigenvalues of the total variance observation scale, extract the sum of the squares of loads. And then we rotate the sum of the squares of the load and the cumulative percentage. In this paper, the cumulative percentage is mainly observed. If it exceeds 50%, it meets the requirement of factor analysis. A total of 5 factors were extracted to explain 93.467% of the total variance. The value is greater than 50%, indicating that the extracted 5 factors can well explain the information contained in the original variables. Item factor load of each dimension is greater than 0.5. And each topic is within the dimensions originally defined, without variable confusion. This shows that the model has high structural validity.

To assess discriminant validity, the squared root of the AVE is expected to be larger than the inter-correlation scores (Li et al., 2020). As shown in Table 3, the square root of AVE on the diagonal is between 0.793 and 0.921, and the absolute maximum value of the correlation coefficient among variables is 0.745, and the minimum value of the square root of AVE is greater than the maximum value of the correlation coefficient, indicating that the scale has good discriminant validity.

**TABLE 3:** Correlation analysis*.

	Average	Standard deviation	HET	CBSH	CONF	OR1	OR2
HET	4.167	1.937	**0.892**				
CBSH	3.672	1.744	0.673**	**0.844**			
CONF	5.081	1.480	−0.541**	−0.370**	**0.793**		
OR1	4.121	1.589	0.690**	0.689**	−0.667**	**0.921**	
OR2	4.964	1.763	0.713**	0.724**	−0.710**	0.745**	**0.883**

*p < 0.05 **p < 0.01; The numbers in bold on the diagonal represent the square root of AVE.

### Correlation analysis

In the correlation analysis of various numerical variables, the commonly used statistical analysis method is Pearson correlation coefficient (Ensley, 1997). Academics use it to measure correlations between things or variables. The academic circle reveals and reflects the correlation between different things or variables through the form of numerical quantification (Omri and Boujelbene, 2021). As can be seen from Table 3, the mean values of entrepreneurial team heterogeneity, cross-border search behavior and organizational resilience are 4.167, 3.672, 4.121, and 4.964, respectively. These values are in the middle. It indicates that the heterogeneity, the level of cross-border search behavior and organizational resilience of entrepreneurial teams need to be improved. The average level of Confucian culture is 5.081, indicating that Confucian culture is at a high level (Aga et al., 2016). All the five variables showed a positive correlation. As shown in Table 3. This provides preliminary support for the above research hypothesis.

### Result

To test the hypotheses, this study has used a moderated mediation model, it is a statistical method that comprises mathematical and statistical approaches for examining data, to identify relationships between variables (Borah et al., 2021). This study has conducted SPSS and PROCESS3.3. The software is useful for measuring mediating and moderating effects and are suitable for the exploratory nature of study analysis. The conceptual framework for this study falls under model 8 of Hayes Model Templates and was estimated as such. In recent years the number of published articles using moderated mediation model increased significantly.

The first hypothesis proposed the heterogeneity of entrepreneurial team (HET) to have a direct positive effect on organizational resilience (OR). From Table 4, we realized that HET had a positive effect on adaptive recovery ability (OR1) and integrated optimization ability (OR2), and these were statistically significant (*0.1545* and *0.1410*). H1a and H1b were, therefore, supported by the analysis.

**TABLE 4:** Table of coefficients (direct effect, moderating effect).

Outcome variable:						

OR1						

Model summary						
***R***	***R*-sq**	**MSE**	***F***	**df1**	**df2**	***p***

0.8315	0.6915	0.7971	94.6249	9.0000	380.0000	0.0000

**Model**						

	**Co eff**	**se**	***t***	***p***	**LLCI**	**ULCI**

Constant	3.014	0.4351	6.9270	0.0000	2.1585	3.8695
HET	0.1545	0.0351	4.3991	0.0000	0.0854	0.2235
CBSH	0.3671	0.0354	10.3836	0.0000	0.2976	0.4367
CONF	−0.4558	0.0378	−12.0458	0.0000	−0.5302	−0.3814
Int_1	0.0235	0.0192	1.2273	0.2205	−0.0142	0.0613
K1	−0.0867	0.0927	−0.9347	0.3505	−0.2690	0.0957
K2	−0.0265	0.0435	−0.6088	0.5430	−0.1121	0.0591
K3	−0.0339	0.1117	−0.3033	0.7618	−0.2536	0.1858
K4	0.0096	0.0491	0.1965	0.8444	−0.0869	0.1061
K5	0.0304	0.0372	0.8164	0.4148	−0.0428	0.1035

**Outcome variable:**						

**OR2**						

**Model summary**						

***R***	***R*-sq**	**MSE**	***F***	**df1**	**df2**	***p***

0.8857	0.7844	0.6860	153.6564	9.0000	380.0000	0.0000

**Model**						

	**Co eff**	**se**	***t***	***p***	**LLCI**	**ULCI**

Constant	3.8468	0.4036	9.5303	0.0000	3.0531	4.6404
HET	0.1410	00326	4.3275	0.0000	0.0769	0.2050
CBSH	0.4302	0.0328	13.1172	0.0000	0.3658	0.4947
CONF	−0.5942	0.0351	−16.9263	0.0000	−0.6632	−0.5252
Int_1	0.1022	0.0178	5.7432	0.0000	0.0672	0.1372
K1	0.0063	0.0860	0.0732	0.9417	−0.1628	0.1754
K2	0.0015	0.0404	0.0370	0.9705	−0.0779	0.0809
K3	−0.0692	0.1037	−0.6673	0.5050	−0.2730	0.1346
K4	−0.0513	0.0455	−1.1269	0.2605	−0.1408	0.0382
K5	0.0102	0.0345	0.2943	0.7687	−0.0577	0.0780

The second hypothesis presented Cross-border search behavior (CBSH) to positively mediate the relationship between HET and OR. From Table 4, we realized that HET had a significant positive effect on CHSH and OR (OR1 and OR2), while the Sobel test presented in Tables 5, 6 showed that the indirect effect was statistically significant, thereby, supporting H2.

**TABLE 5:** Direct and indirect effects of the OR1 and index of moderated mediation.

Conditional direct effect(s) of HET on OR1:						

CONF	Effect	se	*t*	*p*	LLCI	ULCI
−1.8308	0.1114	0.0509	2.1865	0.0294	0.0112	0.2115
−0.0808	0.1526	0.0352	4.3309	0.0000	0.0833	0.2218
1.6692	0.1938	0.0463	4.1860	0.0000	0.1028	0.2848

**Conditional indirect effects of HET on OR1:**						

**Indirect effect:**						

**HET→CBSH→OR1**						

**CONF**	**Effect**	**Boot SE**	**Boot LLCI**	**Boot ULCI**		

−1.8308	0.1819	0.0296	0.1278	0.2429		
−0.0808	0.2165	0.0263	0.1675	0.2690		
1.6692	0.2512	0.0329	0.1917	0.3184		

**Index of moderated mediation:**						

**Index**	**Boot SE**	**Boot LLCI**		**Boot ULCI**		

CONF	0.0198	0.0097	0.0014	0.0395		

**Pairwise contrasts between conditional indirect effects (Effect 1 minus Effect 2)**						

**Effect 1**	**Effect 2**	**Contrast**	**Boot SE**	**Boot LLCI**	**Boot ULCI**	

0.2165	0.1819	0.0346	0.0171	0.0024	0.0691	
0.2512	0.1819	0.0692	0.0341	0.0048	0.1382	
0.2512	0.2165	0.0346	0.0171	0.0024	0.0691	

**TABLE 6:** Direct and indirect effects of the OR2 and index of moderated mediation.

CONF	Effect	se	*t*	*p*	LLCI	ULCI
−1.8308	−0.0462	0.0473	−0.9775	0.3289	−0.1391	0.0467
−0.0808	0.1327	0.0327	4.0609	0.0001	0.0685	0.1970
1.6692	0.3116	0.0429	7.2562	0.0000	0.2272	0.3961

**Conditional indirect effects of HET on OR2:**						

**Indirect effect:**						

**HET→CBSH→OR2**						

**CONF**	**Effect**	**Boot SE**	**Boot LLCI**	**Boot ULCI**		

−1.8308	0.2132	0.0314	0.1552	0.2772		
−0.0808	0.2538	0.0248	0.2062	0.3040		
1.6692	0.2943	0.0317	0.2339	0.3579		

**Index of moderated mediation:**						

**Index**	**Boot SE**	**Boot LLCI**		**Boot ULCI**		

CONF	0.0232	0.0112	0.0016	0.0457		

**Pairwise contrasts between conditional indirect effects (Effect 1 minus Effect 2)**						

**Effect 1**	**Effect 2**	**Contrast**	**Boot SE**	**Boot LLCI**	**Boot ULCI**	

0.2538	0.2132	0.0406	0.0195	0.0028	0.0799	
0.2943	0.2132	0.0811	0.0391	0.0056	0.1598	
0.2943	0.2538	0.0406	0.0195	0.0028	0.0799	

The fourth hypothesis presented Confucian traditional culture (CONF) to moderate the relationship between HET and OR. From Table 5, it can be seen that when Confucian traditional culture is at −1.8308, its moderating effect in this action path (HET→CBSH→OR1) is 0.1819, and Boot LLCI = 0.1278 and Boot ULCI = 0.2429, with 0 excluded between them, indicating that the moderating effect is significant. Similarly, when Confucian culture is at 1.6692, Boot LLCI = 0.1917, Boot ULCI = 0.3184, excluding 0 between the two, so it can be considered that Confucian traditional culture has a positive moderating effect on the mediating effect, which means that when the intensity of Confucian traditional culture is high, the mediating effect of the mediating variable between HET and OR1 will be more powerful. That is, at higher levels of CONF, the mediating effect of CBSH becomes relatively stronger. Using 5,000 bootstrap samples for bias-corrected bootstrap with 95% confidence intervals, Index of Moderated–Moderated–Mediation was statistically significant.

From Table 5, it can be seen that when Confucian traditional culture is at −1.8308, its moderating effect in this action path (HET→CBSH→OR2) is 0.2132, and Boot LLCI = 0.1552 and Boot ULCI = 0.2772, with 0 excluded between them, indicating that the moderating effect is significant. Similarly, when Confucian culture is at 1.6692, Boot LLCI = 0.2339, Boot ULCI = 0.3579, excluding 0 between the two, so it can be considered that Confucian traditional culture has a positive moderating effect on the mediating effect, which means that when the intensity of Confucian traditional culture is high, the mediating effect of the mediating variable between HET and OR2 will be more powerful.

**FIGURE 3:**
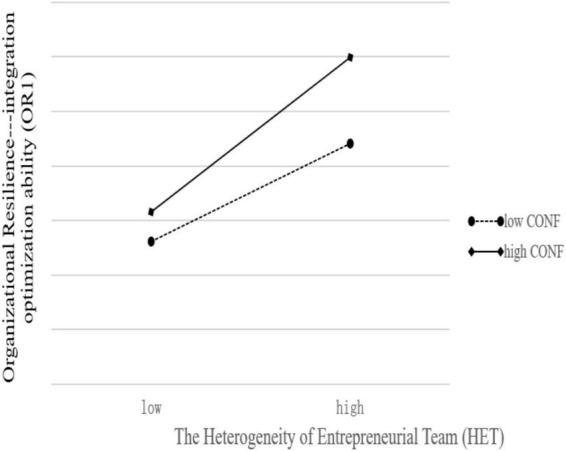
Conditional effects of HET on OR1 at values of CONF.

**FIGURE 4:**
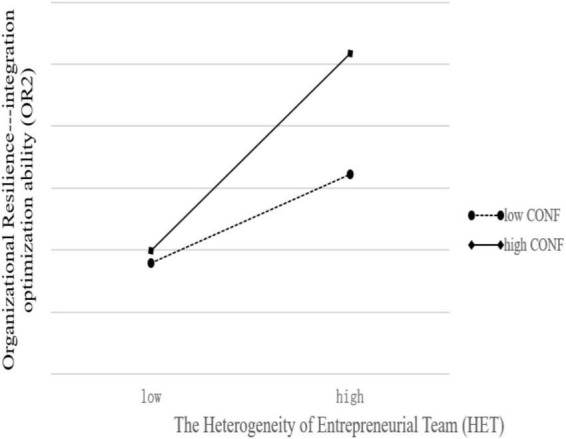
Conditional effects of HET on OR2 at values of CONF (steeper).

Figure 3 showed that, at higher levels of CONF (Solid line), the effect of HET on OR1 becomes greater. Similarly, Figure 4 showed that, at higher levels of CONF (Solid line), the effect of HET on OR2 becomes greater, too. The difference is that the solid line in Figure 4 is steeper than Figure 3. This means that Confucian traditional culture has a stronger and more significant positive moderating effect on the relationship between HET and OR2.

## Discussion

This study has examined the heterogeneity of entrepreneurial team on organizational resilience (Adaptive recovery ability and Integrated optimization ability). It has also analyzed the role of Confucian traditional culture in these relationships. Hypothesis 1 is accepted, showing that the heterogeneity of entrepreneurial team has positive influences on organizational resilience (Adaptive recovery ability and Integrated optimization ability) (Kelly et al., 2021). Hypothesis 2 is also accepted, showing that cross-border search plays a mediating role between the heterogeneity of entrepreneurial team and adaptive recovery ability and integrated optimization ability of the organization. Hypothesis 3 are accepted, which means that Confucian traditional culture has a positive moderating relationship between the heterogeneity of entrepreneurial team and cross-border search. Hypothesis 4 are partly accepted, which mean that Confucian traditional culture has a positive moderating relationship between the heterogeneity of entrepreneurial team and adaptive recovery ability of organization.

Since modern times, the impact of contemporary science and technology and contemporary culture has led to significant changes in the traditional way of production and life (Haryanto et al., 2021; Yee et al., 2021). However, the Confucian thought of settling the land and relocating the ground still profoundly affects the decision-making of enterprises (Payne et al., 2021). Confucian culture, such as Confucian business thought, has been deeply integrated into Chinese enterprise culture (Le et al., 2021). These cultures are reflected in the operating philosophy and management philosophy of Chinese enterprises. As the mother of Chinese culture, Confucian culture permeates every aspect of social and economic development (Lee, 2021), is an unavoidable management rule in a commercial society (Bennett, 2021), and has become a critical spiritual pillar in Chinese modernization (Jiang and Wei, 2021). Confucianism contains a wealth of innovation and entrepreneurship wisdom, such as “unity of knowledge and action,” “empty talk will harm the country, and solid work will prosper the country,” warning entrepreneurial teams to combine theory and practice (Liu et al., 2021). Confucianism also includes the spiritual drive and moral foundation for innovation and entrepreneurship (Eggert and Roetz, 2022).

This study mainly draws the following conclusions: (1) The heterogeneity of entrepreneurial team can promote the formation of organizational resilience; (2) Cross-border search plays a partially mediating role in the relationship between the heterogeneity of entrepreneurial team and organizational resilience, that is, the heterogeneity of entrepreneurial team can promote organizational resilience by promoting organizational cross-border search behavior; (3) Confucian traditional culture positively moderates the relationship between the heterogeneity of entrepreneurial team and organizational resilience, and positively moderates the mediating effect of the heterogeneity of entrepreneurial team on organizational resilience through cross-border search. The more deeply influenced by Confucian culture, the heterogeneity of entrepreneurial team has a more substantial promoting effect on organizational resilience, which is more evident in improving organizational integration and optimization ability. The mediating effect of cross-border search on the heterogeneity of entrepreneurial team and organizational resilience is also positively moderated.

The most interesting finding of this study is that it confirms the two sides of traditional Confucian culture. Table 4 in the manuscript shows that Confucian traditional culture has a significant negative effect on entrepreneurial resilience of the two dimensions (the coefficient of effect is −0.4558 and −0.5942, respectively). However, Tables 5, 6 show another aspect when adjusting the relationship between independent variable (HET) and dependent variable (OR). It shows a positive moderating effect, and the positive effect of the independent variable on the dependent variable is amplified and enhanced in the new ventures with high influence intensity of Confucian traditional culture. This is very interesting. We can guess that the direct negative effect of Confucian cultural strength on organizational entrepreneurial resilience is offset to some extent by its positive moderating effect on the relationship between entrepreneurial team heterogeneity and organizational resilience. This is an interesting finding, and the positive moderating effect of Confucian cultural tradition can be consciously amplified in future enterprise organization and management in order to offset or weaken its negative effect on organizational entrepreneurial resilience. At present, some of the published academic achievements believe that Confucian traditional culture plays a positive role in enterprise organization and management, and some confirm its negative role, but few of them prove that Confucian traditional culture has two sides in organization and management.

The results of this study extend the antecedent mechanism of organizational resilience from a cultural perspective (Percy and Dow, 2021) and effectively explain the current research debate on the effect of team heterogeneity on team performance (Kim et al., 2021). This paper discusses the mediating impact on cross-border search behavior, expands the theoretical application of Confucian traditional culture as a moderating variable (Han, 2022), and effectively responds to the current research initiative of localized management (Percy and Dow, 2021). The results are helpful in guiding new ventures to cultivate organizational resilience in practice (Koh and Ong, 2021).

### Theoretical implications

The theoretical implications of this manuscript include the following aspects. Firstly, Existing studies have explored the positive predictive effect of the heterogeneity of entrepreneurial team on the sustainable development of the organization (Vo and Nguyen, 2021). Existing studies focus on mediating variables involving more static team identity, power distance within the team (Zhao, 2021), team entrepreneurship orientation (Wu et al., 2021), innovation climate (Gu and Liu, 2021), etc. The more dynamic cross-border search behavior in interaction between the organization and the external environment is ignored. Given this, this study proposes and examines the mediating role of cross-border search. This study enriches the intermediate mechanism of the heterogeneity of entrepreneurial team behavior affecting organizational resilience. Secondly, by studying the moderating effect of Confucian traditional culture, it is beneficial to understand the boundary conditions between the heterogeneity of entrepreneurial team and organizational resilience (Ge et al., 2021) and further understand the differential influence mechanism of the heterogeneity of entrepreneurial team on organizational resilience through cross-border search (Pham and Pham, 2021). This manuscript is helpful in expanding the research on traditional Confucian culture and organizational development in China (Park and Rho, 2016). It is of great significance to carry forward Confucian culture and advocate Confucianism to govern enterprises. The government has repeatedly supported promoting traditional Chinese culture and enhanced cultural awareness and confidence. The research conclusions of this paper provide empirical evidence for the Confucian culture in optimizing ventures corporate governance and improving organizational resilience. China is a relevant context here because it is one of the world’s largest developing economies (Eskreis-Winkler et al., 2014). It had a deep-rooted denunciation of private enterprise, which started changing in the 1990s and now the country has a strong private sector that provides multiple opportunities for entrepreneurship (Eskreis-Winkler et al., 2014). However, the country still has a low level of entrepreneurship so research is needed in this area (Fisher et al., 2019). The key research objectives of this study are to explore influence of the heterogeneity of entrepreneurial team on organizational resilience. Moreover, this article also helps in reducing people’s resistance and prejudice against traditional Confucian culture. More importantly, this manuscript provides practical enlightenment on how to use Confucian traditional culture to cultivate and enhance organizational resilience of new ventures in the context of frequent adverse events in China. Secondly, refine the boundary of the impact of the heterogeneity of entrepreneurial team on organizational resilience. This refinement not only reconciles the differences of previous studies, but also to deepen people’s understanding of how the heterogeneity of entrepreneurial team affects organizational resilience.

### Practical implications

This study has provided valuable practical implications as well. Organizational resilience is a potential path-dependent ability and its benefits take a long time to become apparent. Therefore, the cultivation of tissue resilience is also a long-term development and continuous maturation process.

➀ Enterprises should pay attention to the vital role of the heterogeneity of entrepreneurial team in organizational development and promote cross-border search behavior by activating entrepreneurial team fracture to improve organizational resilience. When building entrepreneurial teams, we should pay attention to the collocation of heterogeneity and not be too homogenous to expand social networks. Heterogeneity is conducive to developing the width and new links, increasing the thinking direction and dimension of team members, avoiding the limitation of single thinking, and fostering organizational resilience by strengthening the fracture of entrepreneurial teams in ventures.

According to the Upper Echelons team theory, the cross-border search behavior of new ventures is the result of senior managers’ cognition, judgment and decision. As the CPU of the enterprise, the entrepreneurial team participates in the formulation of major strategic planning and the initiation of actions. The composition and characteristics of entrepreneurial team members affect the final decision and action execution. The core concept to measure the characteristics of entrepreneurial team members is the heterogeneity of entrepreneurial team. Many scholars focus on this and try to analyze the human factors behind corporate behavior. The heterogeneity of entrepreneurial team refers to the differences in age, gender, educational background, functional background, and other demographic characteristics of the founding team of a new venture. It also refers to differences in professional skills such as tenure and industry experience. Some scholars also include the cognitive differences of senior managers and social capital differences such as the relationship network they have into the connotation of the heterogeneity of entrepreneurial team.

➁ Pay attention to cross-border search behavior. Through cross-border search, network embedding, information exchange and other links, different subjects can interact and cooperate, complement each other’s capabilities, and integrate resources. The organization thus accelerates the development, application, and upgrading of technologies and services to improve organizational resilience.

➂ Attach importance to traditional Confucian culture. Confucian culture is the Chinese traditional mainstream culture. Confucian culture has been constantly adjusting, changing and adapting to the economic and social development environment at that time in thousands of years of historical evolution and development. It plays an important role in the historical evolution. The continuation and inheritance of Confucian culture based on relying on agricultural civilization constantly optimizing and refined a unique social management system, which has an essential impact on the way of thinking and behavior of entrepreneurial teams. At the regional level, we can start with cultural construction and strive to enhance the cultural soft power of the regions where new enterprises are located. At the enterprise level, we should attach importance to absorbing and surpassing the essence of Confucian political thought. We also need to fully recognize that carrying forward the excellent Confucian culture can help promote the prosperity and development of Entrepreneurship in China. In this way, we can further promote the national strategy of “mass entrepreneurship and innovation.”

Confucian culture is the main body and essence of Chinese traditional culture and has a far-reaching influence on social and economic development. In this regard, we need to promote core values and firm cultural confidence, eliminate the conflict and prejudice against Confucian cultural values, actively introduce the economic ethics of Confucianism into the management of new enterprises, and cultivate the moral constraints of the combination of “justice” and “profit” and the ethical norms of “careful and independent” self-discipline. But at the same time, we also need to combine the development of contemporary society to constantly clean up and excavate Confucian culture. In addition, we need to strengthen the improvement and innovation of Confucian culture concept, content and form. We should constantly inject new elements into Confucian culture to ensure that it is compatible with the development of The Times. Only through the above efforts can we better practice the core socialist values and find the “key” to the sustainable development of new enterprises.

## Limitations and future research directions

Inevitably, this study has some limitations: (1) With the strength of Confucian traditional culture as a moderating variable, this study explains the contingency effect of Confucian culture on the path of “the heterogeneity of entrepreneurial team-cross border search-organizational resilience.” However, there are still many cultural factors. In the future, we can combine Taoist culture and Buddhist culture. (2) In this manuscript, the total strength of the heterogeneity of entrepreneurial team is calculated based on different the heterogeneity of entrepreneurial team types instead of separately studying the heterogeneity of entrepreneurial team types. In future studies, we can try to further distinguish the different the heterogeneity of entrepreneurial teams. Thus, the action path of organizational resilience of each fracture zone with different attributes can be described in detail. (3) The research object of this paper is new ventures. The heterogeneity of entrepreneurial team is a complex problem and plays a heterogeneous role in developing enterprises. In the context of Localization in China, this study can use a more dynamic perspective to track whether the heterogeneity of entrepreneurial team continues to have a positive impact on corporate resilience by taking each step of enterprise development as the research object. (4) Organizational resilience is a complex problem (Fan, 2021). Future research can try to introduce cross-hierarchical analysis or configuration methods to integrate factors at different levels, such as environment, organization, team, and employee, into the study (Carleo, 2021; Cheng, 2021; Roker, 2021; Zhang, 2021).

## Conclusion

This study has examined the heterogeneity of entrepreneurial team on organizational resilience (Adaptive recovery ability and Integrated optimization ability) in the context of China. The role of Confucian traditional culture, as a moderator, has also been investigated. Data has been obtained from 390 entrepreneurs in in China. All hypotheses have been tested using SPSS&PROCESS 3.3. It has been found that the heterogeneity of entrepreneurial team has positive influences on organizational resilience (Adaptive recovery ability and Integrated optimization ability). Hypothesis 2 is also accepted, showing that cross-border search plays a mediating role between the heterogeneity of entrepreneurial team and adaptive recovery ability and integrated optimization ability of the organization. Hypothesis 3 are accepted, which means that Confucian traditional culture has a positive moderating relationship between the heterogeneity of entrepreneurial team and cross-border search. Hypothesis 4 are partly accepted, which mean that Confucian traditional culture has a positive moderating relationship between the heterogeneity of entrepreneurial team and adaptive recovery ability of organization. This research has made a theoretical contribution by including team structure, cultural environment, team behavior factors in the model. The study has also made an empirical contribution. It has generated multiple theoretical and practical implications. No comprehensive study has investigated the effects of team structure, cultural environment, team behavior factors on team resilience. The results are helpful in understanding the internal mechanism of the heterogeneity of entrepreneurial team affecting organizational resilience. The results suggest that entrepreneurs should take targeted member matching measures during team building to promote cross-boundary search behaviors at the executive level and improve organizational resilience. Faced with profound market changes and severe market challenges, new enterprises should pay more attention to the study, inheritance, and development of Confucian traditional culture. In this way, new enterprises can fully explore the important value implication of Confucian conventional culture in the development of enterprises, enabling enterprises to continue and develop steadily.

## Data availability statement

The original contributions presented in this study are included in the article/supplementary material, further inquiries can be directed to the corresponding author.

## Ethics statement

The studies involving human participants were reviewed and approved by the Nanjing University of Posts and Telecommunications. The participants provided their written informed consent to participate in this study.

## Author contributions

TS contributed to developing the theoretical framework and overall writing of the manuscript. XT contributed to data collection, data analysis, and editing. Both authors contributed to the article and approved the submitted version.
